# Using Virtual Reality to Enhance Food Technology Education

**DOI:** 10.1007/s10798-021-09669-3

**Published:** 2021-05-06

**Authors:** Daniel Gorman, Simon Hoermann, Robert W. Lindeman, Bahareh Shahri

**Affiliations:** 1 Human Interface Technology, Lab NZ, University of Canterbury, Christchurch, New Zealand; 2 School of Product Design, University of Canterbury, Christchurch, New Zealand

**Keywords:** Virtual reality, Education, Technology education, 360 degree video

## Abstract

The use of Virtual Reality (VR) technology combined with 360-degree images and videos provide an opportunity for teachers to bring students into the classroom even when they are located somewhere else. During the COVID-19 lockdown and pandemic, with students across the world forced into home-based learning via remote teaching, a VR classroom shows potential as a tool for adding depth to their learning. The possibility of immersing students in a virtual environment could provide an answer to motivation and engagement issues for today’s students as well as a solution to some of the current constraints faced by teachers. In particular, VR has the potential to increase the time students are able to spend in (virtual) environments that are suitable for teaching and learning practical skills. With the cost of VR equipment reducing rapidly and the increasing quality of virtual experiences, it appears VR is on the tipping-point of becoming a regular part of school programmes.This article outlines the development and testing of a VR Classroom for the delivery of a food-based lesson with middle school students in a New Zealand school. Kitchens are a costly commodity for schools and the obvious health and safety issues make teaching practical cooking skills challenging. With a focus on student engagement and motivation, data is collected from observation of students using the virtual classroom and a post-test survey. Results show that students were highly motivated and perceived the VR classroom as fun to use.

## Introduction

The majority of cooking classes in New Zealand schools are taught at Years 7 and 8^1^ where most students receive compulsory specialist subject teaching including food-based education. A lack of suitable cooking facilities and current teacher shortages are hindering the delivery of practical-based food education (Gorman et al., 2020). The cost of providing and maintaining cooking facilities is significant, and often weekly usage of these facilities is only around 20 h. The rooms are single-purpose spaces and complex to use for teaching other subjects due to health and safety concerns around food contamination and potentially dangerous kitchen equipment. New Zealand has a known teacher shortage, highlighted by a recent survey of 162 secondary schools that showed 40 percent of schools could not fill specialist teaching positions (Long & Moir, 2018). When teaching positions cannot be filled, student numbers reduce, potentially creating a situation where schools are reluctant to spend money on maintaining kitchens that are not used to capacity.

Hence, it is evident that practical food-based education needs support and virtual reality (VR) has the potential to overcome some of the challenges. VR allows users to be immersed in an alternative world, interacting and exploring it as if it is real. VR has become viable in education as a range of affordable options have become available, thus allowing greater access for all students. The potential of VR as a learning environment is promising and according to Bodekaer (2015) it could actually double student achievement. This might be an ambitious target, but VR definitely provides a higher level of immersion than other technologies (such as remote video conferencing), offering huge scope for use in schools and, in particular, in specialist classrooms where the cost of equipment is prohibitive and a teacher’s ability to provide specific one-on-one teaching is limited.

This article outlines the development of a VR Classroom and its consequential evaluation with students, with the aim of exploring the research question: *How can immersive technologies be used to enhance food-based education in New Zealand schools?*

## Background

### Why virtual reality

I had just finished the introductory VR lesson on the HTC Vive when my guide said, “I will show you something really cool, turn around”. I turned around and an elevator door opened, I stepped in and took a ride to the top floor. When the elevator door opened I was 50 storeys up in the air with city scenes and noises below. A plank was sticking out of the door, over the street a long way below. My guide commented, “You probably know what to do now, just walk out and jump!” Surprisingly, as I looked down, I was scared. I tentatively took two steps onto the plank before stopping and saying, “I can’t do it”. My guide responded saying, “Don’t worry just jump,” but despite knowing it wasn’t real, I couldn’t. I had a go at reaching out with my foot to feel beside the plank but I was too scared of losing balance to put it down. I stepped back into the virtual elevator and returned to the ground. (Lead Author – “Visit to VR Room”, 16th May 2018)

The recount above highlights the power and potential of VR to create an environment that is realistic enough that users feel fear. According to Jerald (2016), “virtual reality is defined as a computer-generated digital environment that can be experienced and interacted with as if that environment were real” (p. 9). This feeling of realism is also highlighted by Curcio, Dipace and Norlund (2016), who claim VR is at the highest end of the immersive spectrum, and Burdea (1999), who describes the keys to effective VR as “**I**^**3**^”—3D **I**nteraction, **I**mmersion and **I**magination.

Curcio et al. (2016) claim immersive technologies like VR and augmented reality (AR) can improve “motivation, engagement and critical thinking” (p. 7). VR is considered an effective medium for training and is widely used in areas where the cost of training in real environments is either too expensive or too dangerous, including medicine and the military. Jin and Nakayama (2013), in their VR workshop training test, claimed the VR method was at least as effective as a safety demonstration in the workshop. Smith and Ericson (2009), while examining the use of VR for a fire safety simulation, found students were highly engaged in what the authors describe as, normally, a mundane lesson. Lindgren et al. (2016) compared a physics simulation being completed on a computer to a full body simulation and the results showed the whole-body activity led to significant learning gains. However, in a similar study, Makransky et al. (2017) concluded the students in VR were more immersed, but learned less than their counterparts, claiming VR simulations may be too distracting.

The medium of 360-degree video is comprised of moving images captured simultaneously in all directions from a specified point. On a mobile device, a viewer can look at a scene from that point in all directions by swiping the screen to move the viewing angle. When viewed using a VR headset, the viewer can simply turn and look around as if they were there. With the ability of 360-degree video to give students a greater feeling of presence in the environment than traditional video (Harrington et al., 2018; Johnson, 2018), the ability for 360-degree content to enhance teaching is apparent. Due to increasing quality and reducing cost, 360-degree cameras are now realistic resources for schools. Combining the resulting footage with a cost-effective VR headset makes bringing high quality 360-degree video into the classroom also realistic.

VR can fully immerse a user to the point where they believe they are in a real world and we argue that this immersion can increase student engagement. Using 360-degree images and video, we can create a realistic-looking virtual classroom where students can learn through specific targeted lessons. When viewing a 360-degree video or still image inside a VR headset, the user is immersed into a virtual world that looks similar to the real world, but with one important exclusion; it does not have the distractions of other participants. Harrington et al. (2018) highlight this extra focus in their comparison of participants experiencing video in 360-degree with those watching traditional 2D media. The authors claim the participants in the 360-degree group were significantly more engaged and exhibited lower unrelated thoughts. The following quote highlights some of the positive effects of using 360-degree video: “When reading, I am easily distracted, but this allowed me to learn and experience it in a way that was hard to distract me, so since all my attention was focused I could ‘stand in their shoes’ more” (Johnson, 2018, p. 233). Harrington et al. (2018) claim the use of 360-degree video took the learning from being an abstract experience to one where participants experience “a sense of immersion/presence in the environment” (p. 997). The authors explained that it allowed participants to *experience* and not merely *observe* the surroundings. On top of the higher engagement levels, there is also evidence that learning can be increased through the use of 360-degree training. Lau et al. (2018), in a study into the use of 360-degree video to increase workplace learning behaviour, found that participants showed significantly higher scores in the areas of professional knowledge and problem solving.

The literature also reveals a range of limitations, and one area of concern is how the students will access the technology. The use of potential VR solutions could be restricted, for example, through a lack of access to suitable devices, headsets or fast internet. The process adopted by Johnson, where VR viewers were issued for short-term use, via the library, is a sensible solution; alternatively, with the reduced cost of mobile headsets, it is plausible that a VR headset could well become part of a student’s stationery list (Johnson, 2018). Another problem, especially while the video quality is still developing, is the issue of VR sickness, an effect similar to regular motion sickness. The likeliness of sickness with 360-degree videos can be reduced by keeping the camera stationary while recording content, but this will not prevent illness for all participants. Johnson (2018) suggests having a computer link available for those who struggle with VR sickness, allowing the capacity to view content outside a headset. Whilst this would allow the users to see similar content, not having the heightened presence the VR headset provides might be disappointing for the user.

## Intervention

Following a study into the needs of food-based educators, the concept of a VR Classroom was developed as a way to deliver lessons in health and safety (Gorman et al. 2020). The most common need raised by teachers in the study was to ensure all students could understand and apply food-based hygiene and safety practices. Additionally, all teachers suggested that they would like to use videos to enhance their programmes and provide options for students who miss classes or need to revisit a lesson.

### System

**Fig. 1:**
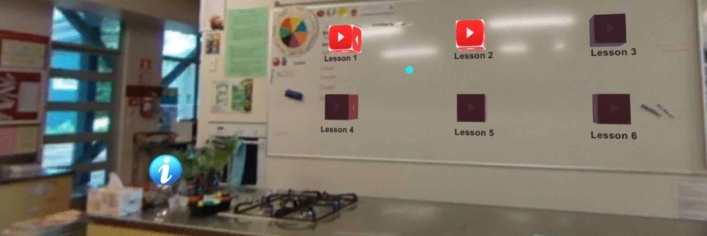
VR Classroom home screen with information hotspots (i) and video links ("play" cubes)

**Fig. 2:**
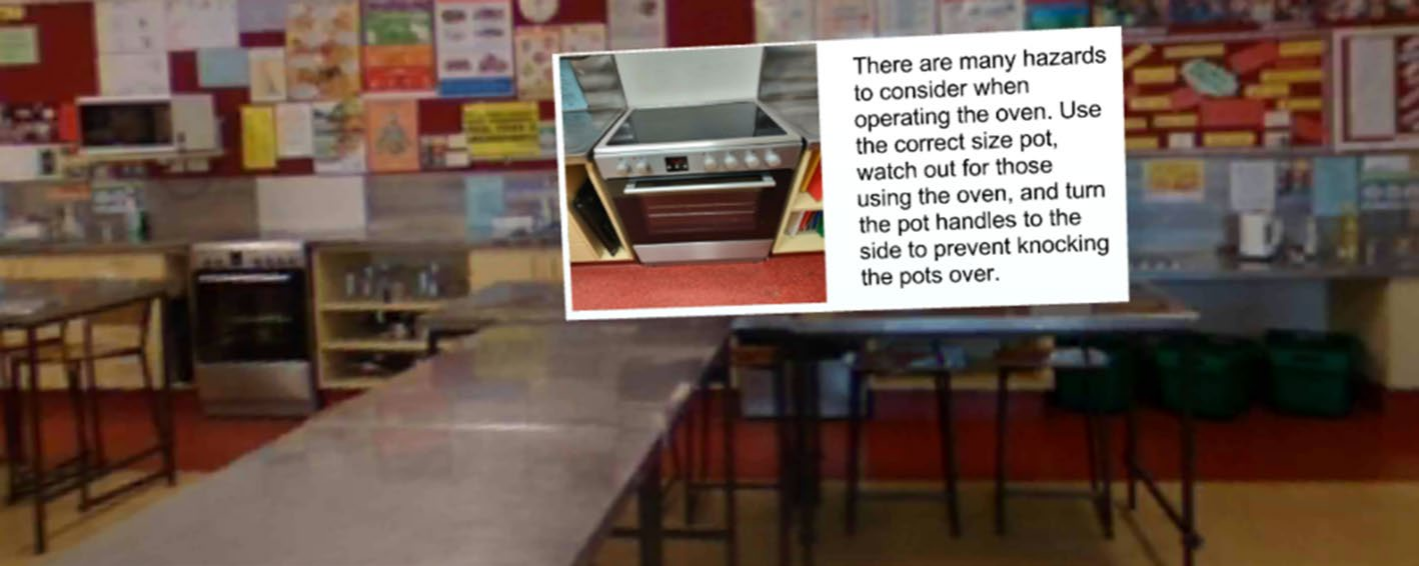
The home screen with the oven hotspot open

Unity (Unity, 2019) was chosen as the platform to develop the VR Classroom because it allows for the greatest number of design elements and provides a resource that can be used beyond this research. The concept of the 360-degree classroom in this research is one that provides food-based education in a virtual environment, allowing students to learn critical health and safety concepts. The design plan (see Fig. 10) was used to guide the design. The interface was built around a 360-degree still image of the school kitchen and contained a range of hotspots that linked to different content. The blue information hotspots and video lesson hotspots are shown in the screenshot of the home screen (Fig. 1). The information hotspots (e.g., Fig. 2) were designed to highlight key learning information, as well as to reveal photos at various key areas of the classroom.

The video icons load different 360-degree video scenes of the teacher demonstrating key health and safety lessons including how to wash hands, cook on the stove, prepare food, wash dishes and sanitise surfaces. Following the videos, a multi-choice question is displayed that allows the students to test their understanding of the tutorial. In order to enhance user understanding, if the multi-choice question is answered correctly, the students receive a positive response and are returned to the home screen, but if the question is answered incorrectly the answer is removed and the user repeats until they get the correct answer.

**Fig. 3:**
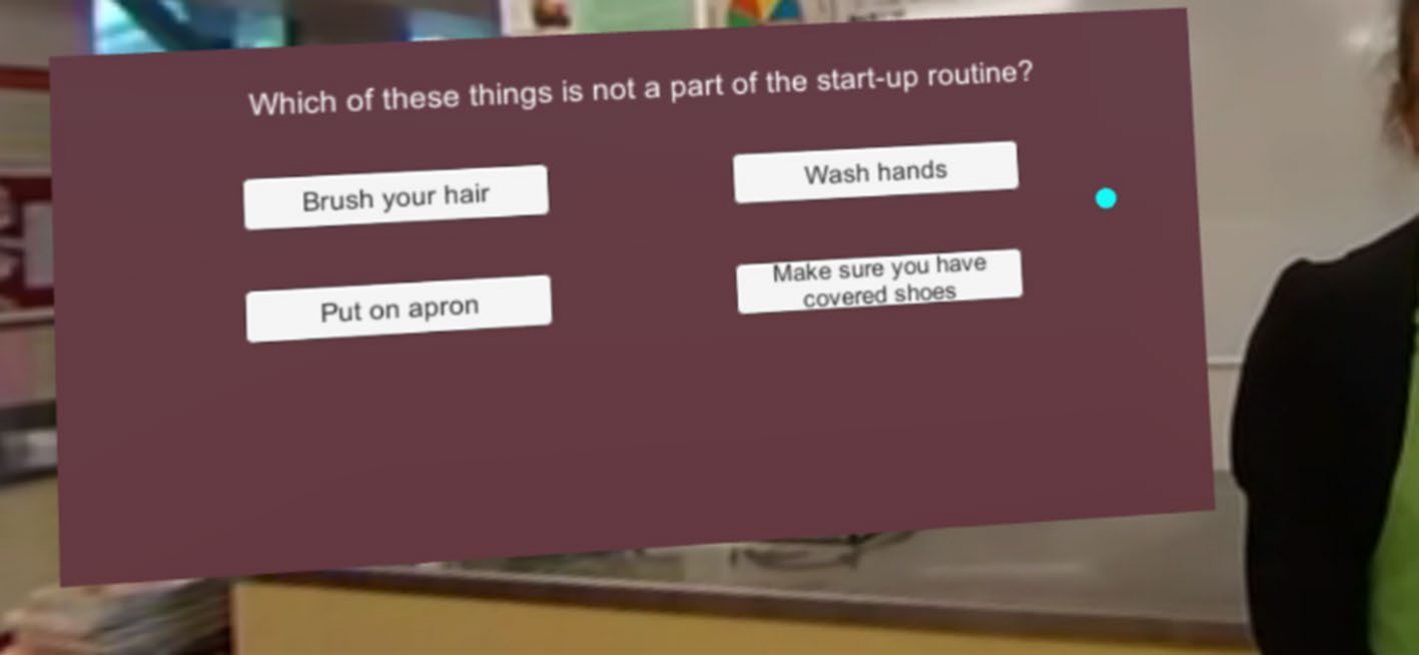
The end of the video for Lesson 1 with the multi-choice video quiz showing

The use of a gaze-click method was adopted as a way to create a product that would be economical for implementation into the classroom via smartphones. The gaze-click method allows the user to interact with and move between screens without the need for a hand controller. When the cursor (the blue dot shown in Fig. 3) is positioned over the trigger point, the cursor grows for three seconds and then opens the link.

**Fig. 4:**
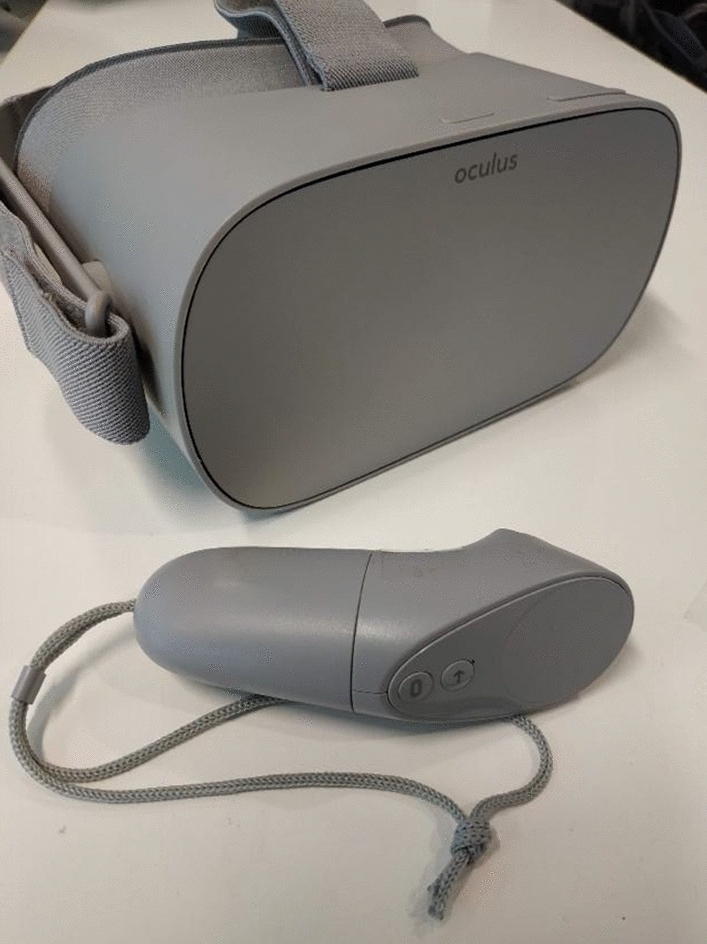
Oculus Go, as used in the user study

The final design was developed for the Oculus Go (Fig. 4), a stand-alone VR viewing device. At a cost of now approximately $150 USD, it is a realistic option for schools and also adds a number of other benefits. It is an all-in-one solution, removing the issues associated with having to use your mobile phone and a separate headset, and offers a high quality video and sound experience. It is easier to develop a VR classroom for Oculus Go devices because the design does not have to be overly generic or specifically adapted for use on several mobile platforms.

One of the key issues in the system development was creating videos that were small enough for the limited storage of the Oculus Go device (32 GB^2^). Initial attempts to reduce the original 360-degree video file size, recorded with our RICOH Theta V camera, resulted in video quality that was poor due to visible compression artefacts. We used Handbrake (HandBrake: Open Source Video Transcoder n.d.), a video transcoder that allowed us to drastically reduce the video file size with only minor visible effects to the video quality using the × 265 video encoder. As an example, one video file was reduced from 696 MB down to 14 MB. Using this method we were able to store all our videos on the device at the same time. We did, however, notice some lag during playback of the video on the devices, which we decided was acceptable for this study. However, in the future we will investigate ways to optimize the trade-off between the size and playback performance of the videos.

## User study

### Method

For this user study we decided to combine user observation and data collected through questionnaires from the potential demographic using the system in an attempt to identify any issues with the design. Tullis and Albert (2013) identify a number of product testing scenarios and one of these is “creating an overall positive user experience” (p. 46). Using this scenario, we collected self-reported and behavioural and physiological metrics on how our participants responded to our system, including monitoring where they were looking and what they were saying. For this study, we recorded comments and noted behaviours observed during the testing. Our self-reported metrics included the System Usability Scale (Brooke, 2013) alongside a series of engagement statements taken from other research publications (see Tables 2 and 3) administrated together as a post-study questionnaire. Each statement in the questionnaire was rated using a 5-point Likert scale to establish levels of agreement:

1. Strongly disagree
2. Disagree
3. Neither agree nor disagree
4. Agree
5. Strongly agree.

### System usability scale (SUS)

We chose to use the all-positive version (Table 1) of the System Usability Scale (SUS), by Sauro and Lewis (2012) who claim that with this model “respondents are less likely to make mistakes when responding, researchers are less likely to make coding errors, and the scores will be similar to the standard SUS” (p. 210). Additionally, we chose this version because the younger age of participants in this study increased the chance of confusion in responses. The SUS is described as measuring “the user’s subjective view of the usability of the system” (Brooke, 2013, p. 33), and this matches with the intent of this study—to obtain the participants emotive responses to using the system. The SUS has been widely used for over 20 years as a quick way to measure usability. It has excellent reliability and concurrent validity with other measures of perceived and objective usability. (Lewis, 2018).

**Table 1:** All-positive SUS as used in this study

Statement number	Statement
1	I think I would like to use the system frequently
2	I found the system to be simple
3	I thought the system was easy to use
4	I think that I could use the system without the support of a technical person
5	I found the various functions in the system were well integrated
6	I thought there was a lot of consistency in the system
7	I would imagine that most people would learn to use the VR Classroom very quickly
8	I found the VR Classroom very intuitive
9	I felt very confident using the VR Classroom
10	I could use the VR Classroom without having to learn anything new

### Engagement and motivation

Engagement, for the purposes of this study, describes the ability of the intervention to encourage the users to explore, interact and learn. Motivation and satisfaction are two areas that are closely related to engagement. Wang (2015), in a study of “Kahoot!” in a university setting, tested for both engagement and motivation, and Barneche et al. (2015), in their study of a virtual museum tour for students in secondary school, used two statements specifically designed to explore satisfaction.

Whilst Barneche et. al. (2015) in their experimental study do not mention validity or reliability, Wang (2015) discusses varied threats to validity and acknowledges the application of their results only for game-based student response systems including Kahoot!. Wang believes the validity would hold if transferred to a similar game-based student response system but not necessarily to a non game based system. Table 2 highlights the statements used by the above authors and shows how they have been adapted for this research.

**Table 2:** Questions 11–17, (Barneche and Hernández 2015, p. 397; Wang 2015, p. 11)

Statement number	Author | Intent	Original statement	Adapted statement
11	Wang (2015) engagement	I was engaged while playing	I was engaged while in the VR Classroom
12	Wang (2015) engagement	It was fun to play the game	It was fun to visit the VR Classroom
13	Wang (2015) motivation	I wish Kahoot! was used in other lectures	I wish we could use this sort of activity in other subjects
14	Wang (2015) motivation	I am more positive towards the topic after playing the game	I would be more positive about foods if we used this technology
15	Barneche et al. (2015) satisfaction	Desire to repeat with another subject	I would like to have another go in the VR Classroom
16	Barneche et al. (2015) satisfaction	General impressions of the experience	Overall I was impressed by the VR Classroom

### Game engagement questionnaire (GEQ)

We also used two statements from the Game Engagement Questionnaire (GEQ) (Brockmyer et al., 2009). This 19-point survey is designed specifically to explore participant engagement in games. The GEQ uses a range of tests with strong reliability and validity data. Whilst the GEQ showed promise as a way of measuring engagement, only some aspects of it, shown in Table 3, were deemed suitable for this study due to the limited testing time and young age of the participants.

**Table 3:** Questions 17–18, (GEQ)

Statement number	Statement
17	It felt real in the VR classroom
18	I lost track of time in the VR classroom

### Procedure

Testing was completed in February 2019. Because the learning contained in the VR Classroom was relevant to the course objectives being covered, the testing was completed as part of the regular classroom programme, simulating one situation for which the product was designed. One class was identified by the classroom teacher as suitable for the test, and all of the students were given written invitations to be a part of the trial. In total, 12 participants—5 male and 7 female—provided consent and completed the trial. Participants were tested in pairs to help reduce potential nervousness. The testing procedure followed the format below:

- A pair of participants were accompanied from the classroom to the adjacent office area by Assistant 1.
- The researcher explained the testing procedure.
- The participants were moved from the office to the testing space and sat on swivel chairs.
- The participants were fitted with an Oculus Go headset.
- The participants completed the following tasks inside the VR Classroom:
- Viewed on the (**i)** (information) spots and read the content.
- Watched ‘video lesson 1’.
- Completed the multi-choice quiz at the end of the video.

6. Participants removed the headset and completed the questionnaire on separate iPads.

## Results

### SUS scores

**Fig. 5:**
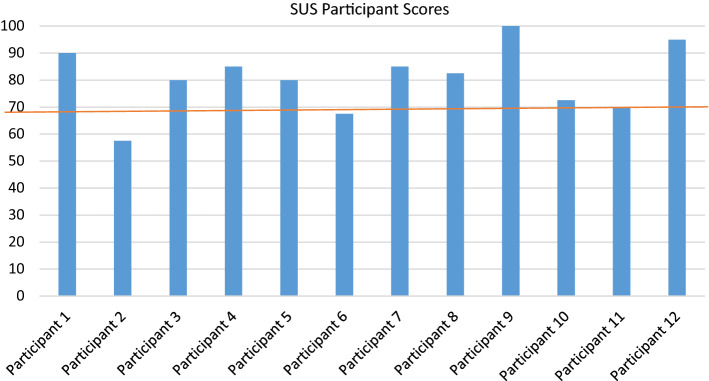
Overall SUS scores

The SUS Score was calculated using the SUS Calculator downloaded from (Tullis, 2018) and adapted to an all-positive format. Figure 5 shows the mean scores of participants graded against the overall mean SUS Score of 68, which is based on thousands of collected datasets (Sauro & Lewis, 2012). This chart shows that 10 out of 12 participants ranked the VR Classroom as better than the established average of 68.

As of yet, there is no benchmark data for SUS completed with VR; therefore, we used the overall mean for this discussion. That said, in a recent study comparing the Oculus Rift and Samsung Gear VR, Webster and Dues (2018) reported SUS means of 82.7 and 82.9 (p. 8), respectively, much higher than the established overall mean of 68. Therefore, it is likely that the benchmark mean for VR could be higher than 68. Nevertheless, the mean score of 80.4 achieved in our study suggests that the system is very usable and is in alignment with results achieved by successful interactive tools like iPhone at 78.5 or the Wii at 76.9 (Sauro & Lewis, 2012, p. 205).

### SUS by gender

**Fig. 6:**
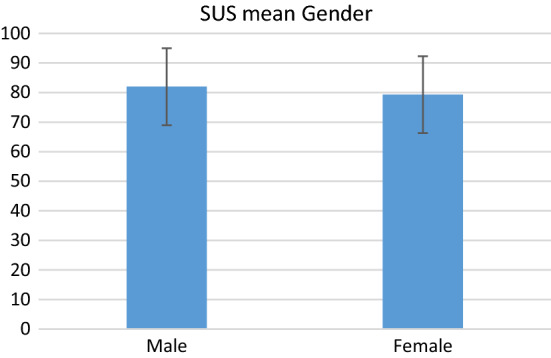
Male vs female SUS with confidence

When analysing SUS data by gender, the mean score of the five male and seven female participants is similar, 82 and 79.3 respectively. There is no significant difference, at α = 0.05, between the mean SUS scores of male and female participants shown in Fig. 6 (two-tailed *t*-test *p* = 0.720). An *F* test, to determine if the variance between genders was different, was also not significant at (*p* = *0*.520). There is a larger range in the scores for female participants, with a difference of 42.5 (57.5–100) compared to 27.5 (67.5–95) from male respondents.

### Engagement

**Fig. 7:**
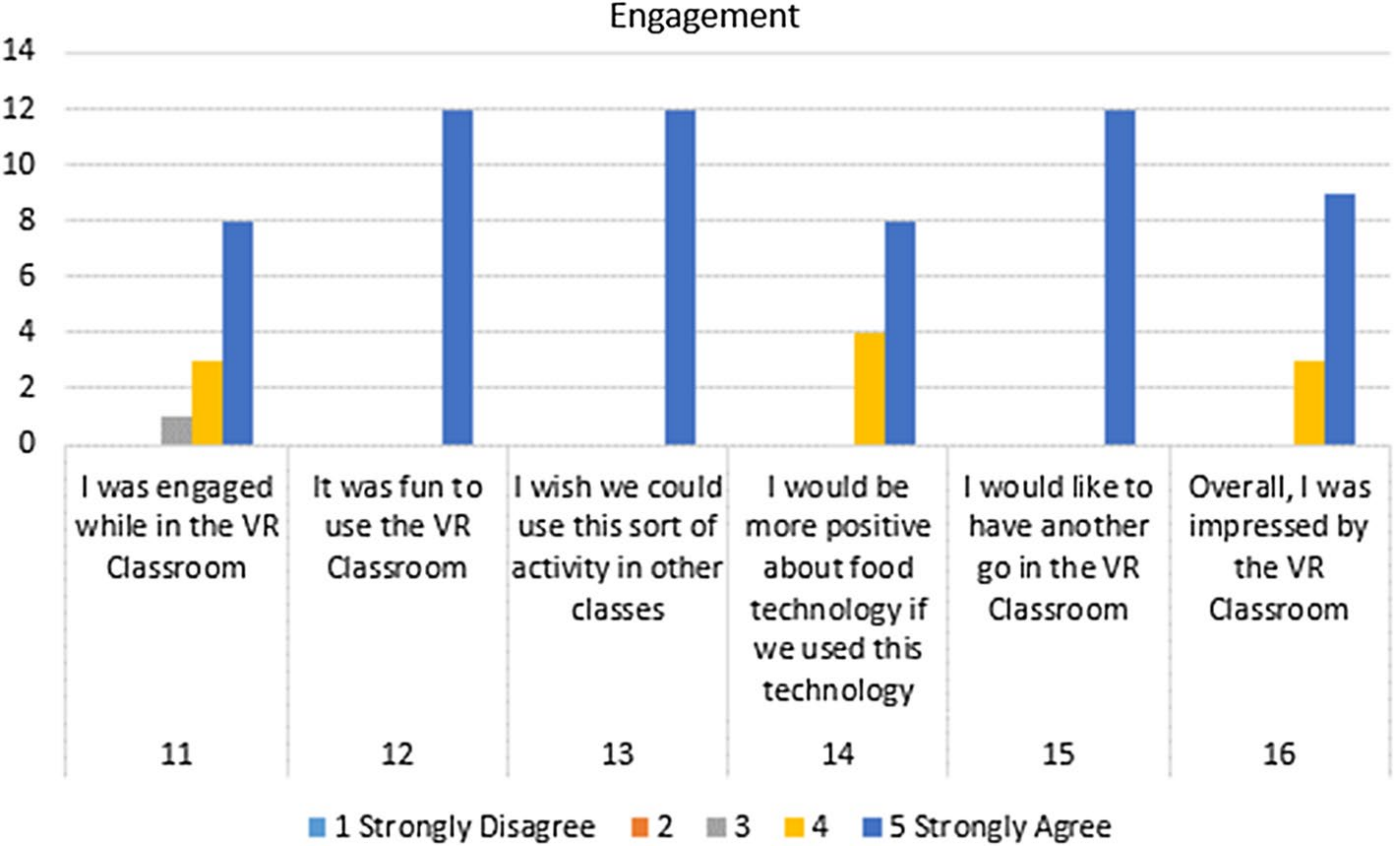
Engagement statement responses

Statements 11–16 targeted motivation and engagement. Figure 7 highlights a very positive response from participants. Statements 12, 13 and 15 received the highest possible score from all participants. Statement 11 (“I was engaged while in the VR Classroom”) was the only statement in this section to receive a response lower than “Agree,” with one participant selecting neutral. All of the responses for Statement 14 and 16 were either “Agree” or “Strongly Agree.”

**Fig. 8:**
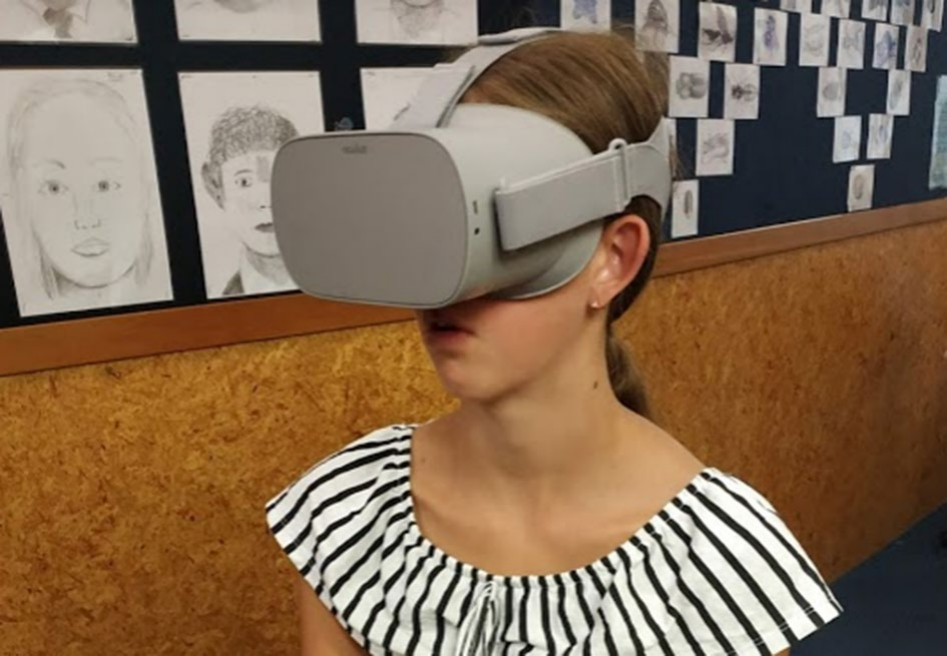
Testing the VR classroom

During the experiment, we noted participants’ impromptu comments that supported interest in the VR Classroom. Three participants said “Wow” soon after putting the headset on, and two of them also added “This is cool”. The predominant behaviour observed was a high level of focus with participants calmly viewing the different aspects of the virtual classroom, similar to that shown in Fig. 8. One boy, however, was particularly excited and spent a good portion of time spinning on the chair while looking up and down to explore the entire virtual space.

### Game experience questionnaire (GEQ)

**Fig. 9:**
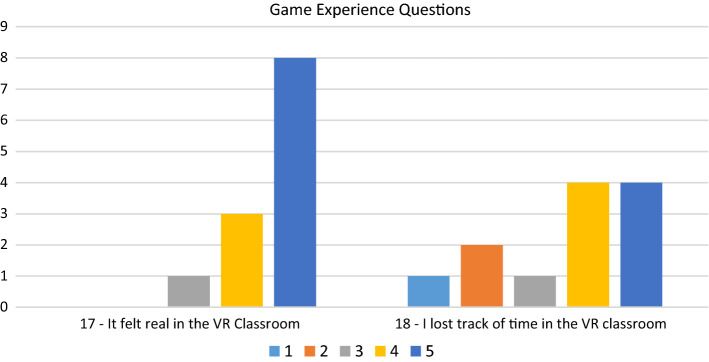
Game experience questions

**Fig. 10:**
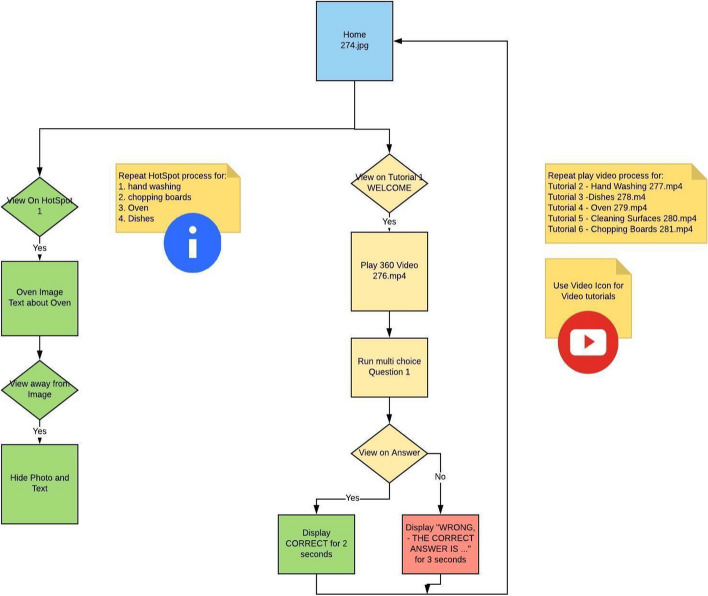
Design flow chart for 360-degree classroom

The two statements from the game experience questionnaire received slightly lower ratings. Nevertheless, as shown in Fig. 9, eight out of the 12 participants still strongly agreed with Statement 17 (“it felt real in the VR Classroom”). The slightly lower rating is supported by two unexpected observations. Two students commented on how strange it was looking down, indicating they felt like they were up in the air. We also observed one participant jump saying “UGH, it’s (teacher’s name)” when the tutorial video started and there was suddenly a person in the classroom with them.

Statement 18 (“I lost track of time in the VR Classroom”) was the lowest scored statement in the questionnaire with a wider range of ratings.

## Discussion

### Engagement

This research specifically asked: *How can immersive technologies be used to enhance food-based education?* The results give us some insights into the potential of the VR Classroom tested. We specifically asked participants to rate their agreement with the statement: *I was engaged while using the VR Classroom,* and eight out of 12 participants strongly agreed, three agreed and one responded as neutral. This was the lowest ranked of the six statements in the engagement section, and matches observational data that reported that four participants were moving and talking during the VR part of the experiment. This offers some support to the findings of Makransky et al. (2017) that VR could be too distracting for some learners. Nevertheless, we think even these students were engaged; it was just that their focus, exploring the environment, was different from the intent of the test. In future versions, we propose that the participants be given a question, prior to the test, that encourages them to watch and engage with the content in the VR Classroom. Their focus could also be increased through the addition of game elements like badges and points in the final product. To gain more accurate responses and minimise distraction during testing, we propose allowing time to explore the environment prior to completing the specified testing tasks.

The other statements in the engagement section of the questionnaire, strongly support the fact that students enjoyed the experience and the potential of the VR Classroom to engage. One hundred percent of participants strongly agreed that it was fun (Statement 12), wanted to use this sort of activity in other classes (Statement 13), and wanted to have another go (Statement 15). In particular, Statement 1 of the SUS asks if participants would like to use this type of system more frequently, and when scored individually this statement received a score of 90. Thomas (2015) proposes that a mean SUS score of 80.3 is the critical point where users will tell their friends about your product. Our SUS Score of 80.4 and the anecdote below, recorded 15 min after the completion of the test, supports Thomas’ notion and highlights the potential of this type of solution to capture students’ attention:

Having finished packing up after the testing, the researcher walked across to thank the classroom teacher, who had just finished her end of day duty by the road. The teacher explained how one of the students—not part of the user study class—had come running up to them saying, (as they switched to an imitation of an excited child’s voice) "I heard that you were doing some cool VR thing in your room and it was really cool. Can we do this in our class too? (Research notes 19 February 2019).

Some problems in the design of the VR Classroom affected the overall engagement, but also revealed a sense of realism in the experience. We noted twice that participants had concerns about the distance to the floor. In review, it was because the 360-degree images and videos were recorded with the tripod set to an average height of a 12-year-old student in a standing position, approximately 140 cm off the ground. We, however, decided to test using a swivel chair to take away any risk of falling or walking into objects while testing. Consequently, in the VR Classroom the approximate eye height of participants was around 30 cm below the video height, meaning that when they viewed the floor inside the VR headset it was far lower than their senses told them it should be. This effect was a distraction for some students, but, conversely, the fact that they found it unsettling meant that they were experiencing a sense of realism, displaying a response similar to the researcher who was afraid to put their foot down in the lift simulation. The importance of eye-height in VR was also demonstrated by Zhang et al. who found that the perspective from which VR simulations are experienced influences how risks are perceived in a virtual environment (Zhang et al., 2020).

A second observation suggesting that students experienced a strong sense of presence was when one participant spoke in a surprised voice, as if they had been given a fright, saying “Ugh, it’s (teachers’ name)” when the teacher appeared in the room as the tutorial video started. The participant was surprised to find a person moving in the VR classroom, effectively in the room with them. Finding a way to fade the video in over the still image could provide a solution that allows the characters to arrive in the VR space without surprising the viewers.

### Limitations

It is possible that the age of our participants, around 12 years old, could have affected the reliability of the data. Comprehension of questions was one of our concerns prior to the test so the questionnaire was shared with the classroom teacher prior to the testing. The teacher was confident that the students had high enough levels of literacy and that the wording was appropriate. The statements in the SUS can be confusing for adults as well as children, but the simplicity of administration, its suitability for use with small sample sizes, its reliability at being able to distinguish levels of usability as well as the reduced confusion provided by the all-positive variant made it suitable for our research (System Usability Scale (SUS) n.d.).

Younger participants might also be less objective in their responses than adults. The ratio of scores in each of the levels of agreement do show a positive trend to responses. However, we argue that the established overall mean SUS score of 68 indicates that adults are also more likely to give positive responses. Additionally, the overall mean of the SUS was consistent with other high immersive studies as described by Webster and Dues (2018). The students showed they could be objective with Statement 18 (“I lost track of time in the VR Classroom”) which shows a wide spread of responses across all areas of the five-point scale. When reviewed, it makes sense that the participants would have mixed responses to this statement, because it was initially designed for participants after many hours playing a game, whereas each participant in this study was only using the VR Classroom for a few minutes.

### Testing procedure improvements

A number of things could be improved in future experiments. At times participants and research assistants were talking during the testing, breaking the immersion of the VR Classroom. For future experiments, we recommend testing is completed individually or in separate sound-proof booths. Video recording the sessions would be beneficial to allow accurate time-sampling of behaviours and enable a full transcript to be prepared. Gaze tracking, technology that tracks the participants’ eyes, could also be useful. However, a video recording of participants alongside a screen capture of what they are seeing would also allow tracking of viewing patterns. Whilst the questionnaire provided a range of post-testing data, a series of post-test interview questions or a focus group to attain participants’ feedback could add depth to the data.

### Future work

While the VR Classroom in this study showed a high level of motivation from participants, the question remains, is it predominantly the novelty of the VR that was motivating? To test this, a study similar to Wang (2015) could be conducted to see if there is a wear-out effect with VR. Participants in two similar sample groups could be exposed to the VR Classroom in different ways. One group having weekly use of the VR Classroom while the other only using it 2–3 times over the duration of the course to see if motivation remains after multiple uses. Because this study was designed to test useability, and in particular motivation, the student response data inside the VR Classroom was not gathered. Future work could collect data from the multi-choice quiz within the VR Classroom and compare this to similar data attained from the other sample group.

The VR Classroom has the potential to remove regular distractions from the classroom that can make learning difficult, such as, a game of football that starts outside the classroom window or students who are being disruptive. On the other hand, adding students into the virtual environment would make it both more realistic and give an opportunity to add student questions as a way of highlighting key learning. We propose a study that explores the inclusion of other characters in the virtual classroom and the effect of this on the user's focus and learning.

A virtual classroom has potential to support inclusive education and this includes a lot of potential areas for future work. Firstly, unlike a regular food lesson that relies on whole class demonstrations, the learning in a VR Classroom can be guided by the students’ needs and students can choose which virtual lessons to attend as they need them. Secondly, at a time when schools are being redeveloped or built with large open-plan classrooms, it is a common concern that some students will struggle in these often noisier environments. The use of a VR Classroom could provide a sanctuary for students that removes some of the noise and distractions and allows them to focus on their learning. Thirdly, for students operating at different levels of the autism spectrum, change can be stressful (Fuld, 2018). The ability to complete lessons in a VR Classroom could allow them to become familiar with new spaces and/or teachers before attending the class. Lastly, at a time when much of the world’s school systems have been shut down, students could watch virtual lessons and be part of a class as if they were there.

## Conclusion

The research question asks how immersive technologies can be used to enhance food-based education in New Zealand secondary schools. We created and evaluated a VR Classroom that uses a range of tools to teach food safety messages. The positive response to the system highlights strong potential to motivate students, and participants’ strong agreement that it was fun, their desire to use the system again and interest in incorporating similar technology into other subject areas highlights the potential of this type of technology to foster engagement.

At a time when much of the world is in lockdown and students are learning from their homes, VR has the potential to support learning and transform educational delivery systems. The ability to join a class and engage with a teacher in a virtual environment would make a novel and effective addition to the typical home programme and allow students to be more fully engaged in the lesson. In face-to-face classrooms VR has the potential to provide quality virtual lessons, freeing up time for teachers to cater to a wider range of individual learning needs. Regardless of the future of education, 360-degree video and images provide exciting potential for today’s educators and learners. The increasing ease with which a teacher can record high quality 360-degree content and distribute it for students, combined with the enormous potential of VR to engage learners, should motivate teachers to embrace this technology and make VR education a reality.

## Data Availability

Data transparency.

## Appendix

See Fig. 10.

## Footnotes

1 Year 7 and 8 in New Zealand schools is usually for students aged between 11 and 13 years old. These students are sometimes referred to as intermediate because an Intermediate school in New Zealand is a form of middle school that only caters to students in Years 7 and 8.

2 Note: Oculus Go is also available in a 64 GB version.

## Code availability

Software application or custom code.
